# Promoting CO_2_ Electroreduction to Acetate by an Amine-Terminal, Dendrimer-Functionalized Cu Catalyst

**DOI:** 10.1021/acscentsci.3c00826

**Published:** 2023-09-26

**Authors:** Li Yang, Ximeng Lv, Chen Peng, Shuyi Kong, Fuqiang Huang, Yi Tang, Lijuan Zhang, Gengfeng Zheng

**Affiliations:** † Laboratory of Advanced Materials, Department of Chemistry and Shanghai Key Laboratory of Molecular Catalysis and Innovative Materials, Fudan University, Shanghai 200438, China; ‡ State Key Laboratory of High Performance Ceramics and Superfine Microstructure, Shanghai Institute of Ceramics, Chinese Academy of Sciences, Shanghai 200050, China

## Abstract

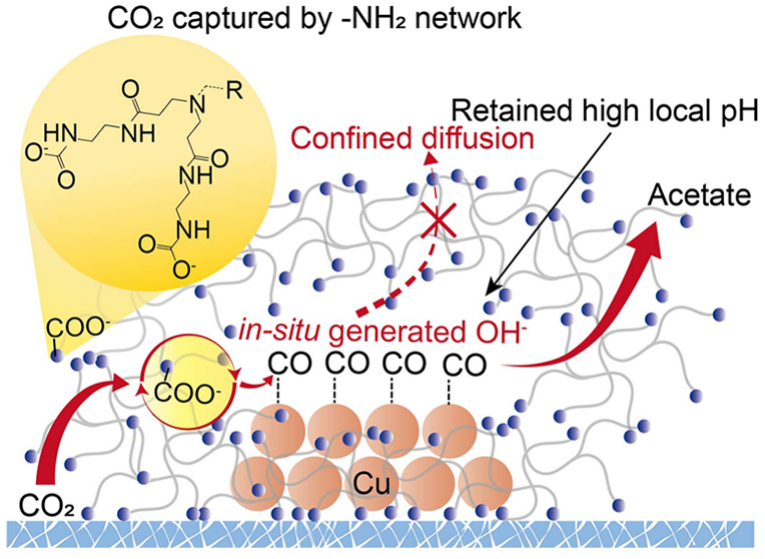

Acetate derived from electrocatalytic CO_2_ reduction represents a potential low-carbon synthesis approach. However, the CO_2_-to-acetate activity and selectivity are largely inhibited by the low surface coverage of *in situ* generated *CO, as well as the inefficient ethenone intermediate formation due to the side reaction between CO_2_ and alkaline electrolytes. Tuning catalyst microenvironments by chemical modification of the catalyst surface is a potential strategy to enhance CO_2_ capture and increase local *CO concentrations, while it also increases the selectivity of side reduction products, such as methane or ethylene. To solve this challenge, herein, we developed a hydrophilic amine-tailed, dendrimer network with enhanced *CO intermediate coverage on Cu catalytic sites while at the same time retaining the *in situ* generated OH^–^ as a high local pH environment that favors the ethenone intermediate toward acetate. The optimized amine-network coordinated Cu catalyst (G_3_-NH_2_/Cu) exhibits one of the highest CO_2_-to-acetate Faradaic efficiencies of 47.0% with a partial current density of 202 mA cm^–2^ at −0.97 V versus the reversible hydrogen electrode.

## Abstract 2

A highly dendritic network with amine terminals was functionalized on Cu nanoparticles as an efficient electrocatalytic CO_2_ reduction catalyst, which was beneficial for enhancing the *CO intermediate coverage and retaining the *in situ* generated OH^−^ on the Cu catalytic sites, thus promoting efficient electrocatalytic CO_2_-to-acetate conversion.

## Introduction

Acetate is an important chemical widely used in manufacturing, medicine, and food. The commercial acetate production is mainly based on the thermal carbonylation of methanol and carbon monoxide (CO), which results in a substantial carbon footprint.^1,2^ The recent development of CO electroreduction has featured a potential alternative means of producing acetate, with a reported acetate Faradaic efficiency (FE) of >70% and partial current density (|*j*_acetate_|) over 425 mA cm^–2^.^3^ On the other hand, although the direct carbon dioxide reduction reaction (CO_2_RR) using renewable electricity has the potential of both reducing greenhouse emission and generating value-added chemicals, the selective CO_2_-to-acetate (or acetic acid) conversion has received only limited progress.

Previous studies in CO electroreduction have suggested that the critical factors of producing acetate include a high coverage of *CO adsorbates on the catalyst surface as well as the formation of ethenone intermediate (*H_2_CCO) under high alkalinity.^3^ Nonetheless, in CO_2_RR, the *CO intermediate is *in situ* generated from a CO_2_ source, and thus its coverage is generally lower than the direct use of CO reactant.^4^ In addition, the side reaction between CO_2_ and the high alkaline electrolyte also results in the fast depletion of CO_2_ molecules and generation of (bi)carbonate that can gradually deactivate the catalyst sites,^5^ further inhibiting the retention of high surface *CO coverage. Even when optimizing the Cu-based electrocatalysts to achieve highest multicarbon (C_2+_) product selectivities of >80%, such as confinement Cu structures^6,7^ or metal-doped Cu oxides^8,9^ to enhance the binding with *CO intermediates, the main C_2+_ products are ethylene^7,8^ and ethanol.^6,9^ To date, the highest partial current density of producing acetate from CO_2_RR is less than 50 mA cm^–2^.^10^

Chemical modification of the catalyst surface has recently been investigated to enhance CO_2_ capture and increase surface *CO coverage.^11−13^ Nonetheless, as *CO is a shared critical intermediate for most of the CO_2_RR products, the scaling relation between different reaction pathways poses a critical limit on the selectivity tuning. For instance, coating of hydrophilic molecules such as polyamides or amino acids on the Cu surface for CO_2_ capture also increases the *H coverage on the catalytic sites, which not only promotes the competitive hydrogen evolution reaction^14^ but also can facilitate the formation pathway of the *CHO intermediate toward CH_4_.^15^ On the other hand, coating of Cu with hydrophobic molecules for increasing local alkalinity can inevitably increase the (bi)carbonate formation on the surface, thus restricting *CO coverage and resulting in preferable C_2_H_4_ selectivity.^16−18^ Thus, it places a challenging dilemma of both increasing *CO coverage and enhancing the acetate pathway.

**Figure 1:**
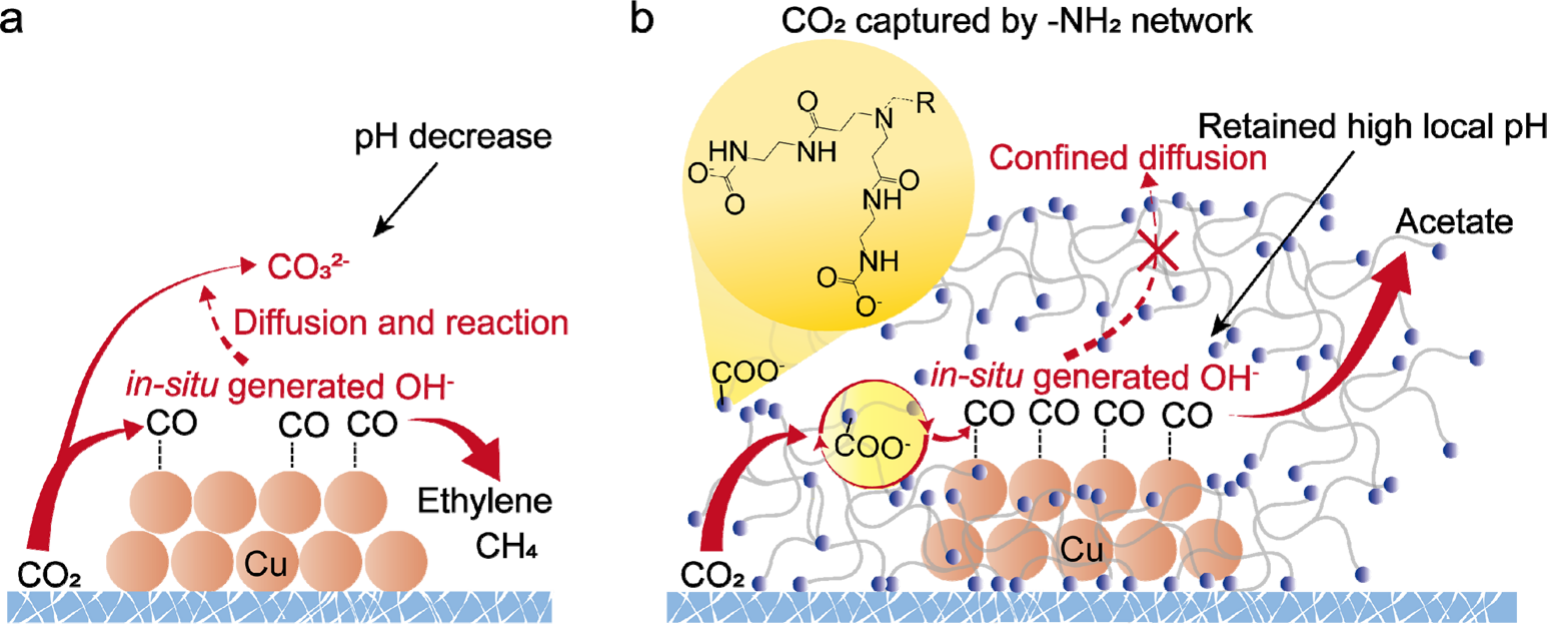
Schematic of the CO_2_RR process on (a) Cu and (b) -NH_2_-terminal, dendrimer-functionalized Cu. The abundant -NH_2_-terminals of the dendritic polymer provide a highly intertwined network to capture CO_2_ for high *CO coverage on Cu and allow strong coordination with the Cu surface to retain the *in situ* generated OH^–^, thus favoring the CO_2_-to-acetate selectivity.

To address this dilemma, we propose that a hydrophilic network can be beneficial for the high coverage of *CO on the catalytic sites while retaining the *in situ* generated OH^–^ to sustain a high local pH environment that favors the *H_2_CCO intermediate toward acetate. Herein, we developed a Cu nanoparticle catalyst functionalized with a highly dendritic polymer with amine (-NH_2_) tails, designated as G_3_-NH_2_/Cu, as an efficient CO_2_-to-acetate electrocatalyst. Compared to pristine (bare) Cu (Figure 1a), the abundant NH_2_-containing tail chain of the dendritic polymer exhibited a highly intertwined network to capture CO_2_ toward higher *CO coverage on Cu, and in the meantime allowed strong coordination with the Cu surface to reserve the *in situ* generated OH^–^, thus maintaining a stable high local pH (Figure 1b). The dendritic polymer-coated Cu electrocatalyst enabled an outstanding CO_2_-to-acetate performance, including one of the highest acetate partial current densities (|*j*_acetate_|) of 202 ± 14 mA cm^–2^ with a corresponding Faradaic efficiency (FE_acetate_) of 47.0 ± 3.1% at −0.97 V versus the reversible hydrogen electrode (vs RHE), suggesting an attractive strategy of surface molecular engineering to tune the scaling relation of different CO_2_RR products.

## Results and Discussion

### Synthesis and Characterizations of Catalysts

The dendrimer was synthesized via a typical divergent method (Methods in Supporting Information).^19^ The degree of crossing-linking dendritic branches and functional terminals was controlled by the number of repeated synthesis cycles (Figure S1a), including full generations of -NH_2_-terminated dendrimers (i.e., G_1_-NH_2_, G_2_-NH_2_, G_3_-NH_2_, G_4_-NH_2_) and half generation of the -OCH_3_-terminated dendrimer (i.e., G_3_-OCH_3_, Figure S1b). Fourier transform infrared (FT-IR) spectra (Figure S2) of both G_3_-NH_2_ and G_3_-OCH_3_ samples showed typical vibrations of N–C=O bonds (1650 and 1557 cm^–1^) and amide-linked -CH_2_- (2834, 2838, and 2927 cm^–1^), assigned to the dendrimer branches.^20−22^ The G_3_-NH_2_ sample showed a broad vibration of -NH_2_ (∼3285 cm^–1^),^22^ while the G_3_-OCH_3_ showed vibrations of -COOCH_3_ (2958, 1735, and 1443 cm^–1^) and C–C=O bond (1362, 1358, 1328, 1048, and 846 cm^–1^).^22,23^

**Figure 2:**
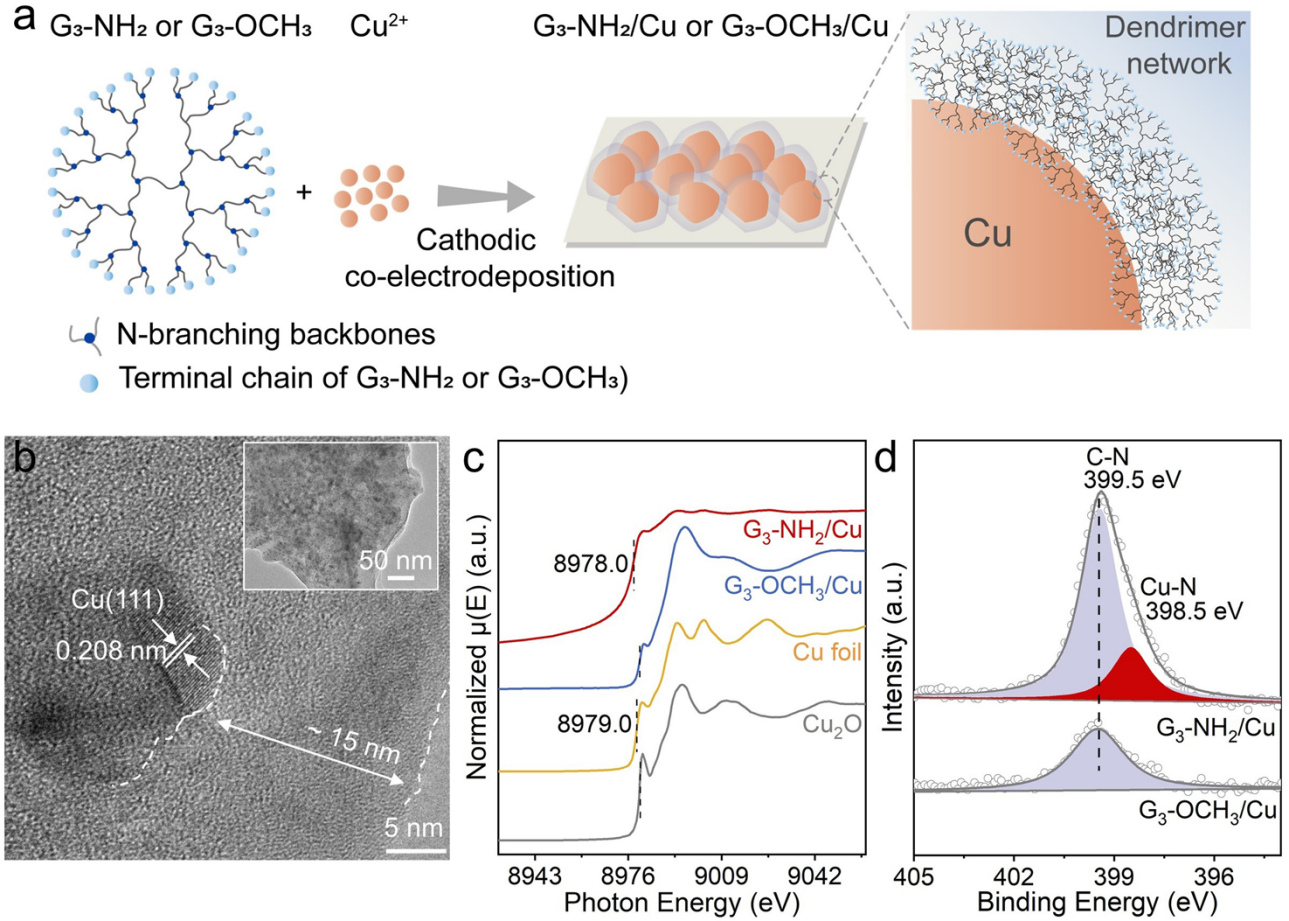
(a) Schematic of co-electrodeposition of -NH_2_-terminal dendrimers and Cu. (b) TEM (inset) and HRTEM images of G_3_-NH_2_/Cu. (c) Normalized Cu K-edge XANES spectra of the G_3_-NH_2_/Cu, G_3_-OCH_3_/Cu, Cu foil, and Cu_2_O samples. (d) N 1s XPS spectra of the G_3_-NH_2_/Cu and G_3_-OCH_3_/Cu.

Those synthesized dendrimers were then functionalized onto the Cu nanoparticles via an electrochemical deposition approach (Methods in Supporting Information, Figure 2a), and they were designated as G_3_-NH_2_/Cu and G_3_-OCH_3_/Cu, respectively. X-ray diffraction of all the samples showed the dominant Cu(111) diffraction at 2θ of 43.3° (JCPDS No. 04-0836, Figure S3). Transmission electron microscopy (TEM) and high-resolution TEM (HRTEM) images displayed the crystalline nanoparticles with diameters of 10–20 nm and lattice spacing of ∼0.21 nm, wrapped in amorphous films of ∼15 nm thickness (Figure 2b and Figure S4). The energy dispersive X-ray spectroscopy (EDS) elemental mapping images showed that Cu, C, O, and N were evenly distributed in both samples (Figures S5 and S6). The obtained EDS atomic N/Cu ratios in G_3_-NH_2_/Cu and G_3_-OCH_3_/Cu were around 0.70 and 0.32, respectively (Table S1), similar to the X-ray photoelectron spectroscopy (XPS) data (Table S2). The metal contents of these samples were verified by inductively coupled plasma analysis (Table S3). The contact angles of G_3_-NH_2_/Cu, G_3_-OCH_3_/Cu, and Cu were measured as ∼87°, ∼98°, and ∼145°, respectively (Figure S7).

The X-ray absorption spectroscopy of the Cu K-edge was then performed to investigate the chemical state and coordination structure. The X-ray absorption near-edge fine structure (XANES) spectroscopy of Cu K-edge in G_3_-NH_2_/Cu and G_3_-OCH_3_/Cu showed different shapes of the rising edge and the post edge (Figure 2c), suggesting their different chemical states and local coordination environments.^24^ The first edge absorption (the first inflection point, black dash in Figure 2c) of G_3_-NH_2_/Cu (∼8978.0 eV) was lower than the Cu foil (∼8979.0 eV),^25,26^ suggesting the electron transfer from the coordination atoms to Cu in G_3_-NH_2_/Cu. The N 1s XPS spectra were recorded to investigate the chemical states of N-containing groups (Figure 2d). The G_3_-OCH_3_/Cu sample retained a C–N bond (∼399.5 eV), while the N 1s XPS spectrum of G_3_-NH_2_/Cu was deconvoluted into two sub-peaks centered at 399.5 and 398.5 eV, ascribed to C–N and Cu–N interactions, respectively,^27,28^ suggesting the electron transfer from N to Cu in G_3_-NH_2_/Cu.

### Electrochemical CO_2_RR Measurements

**Figure 3:**
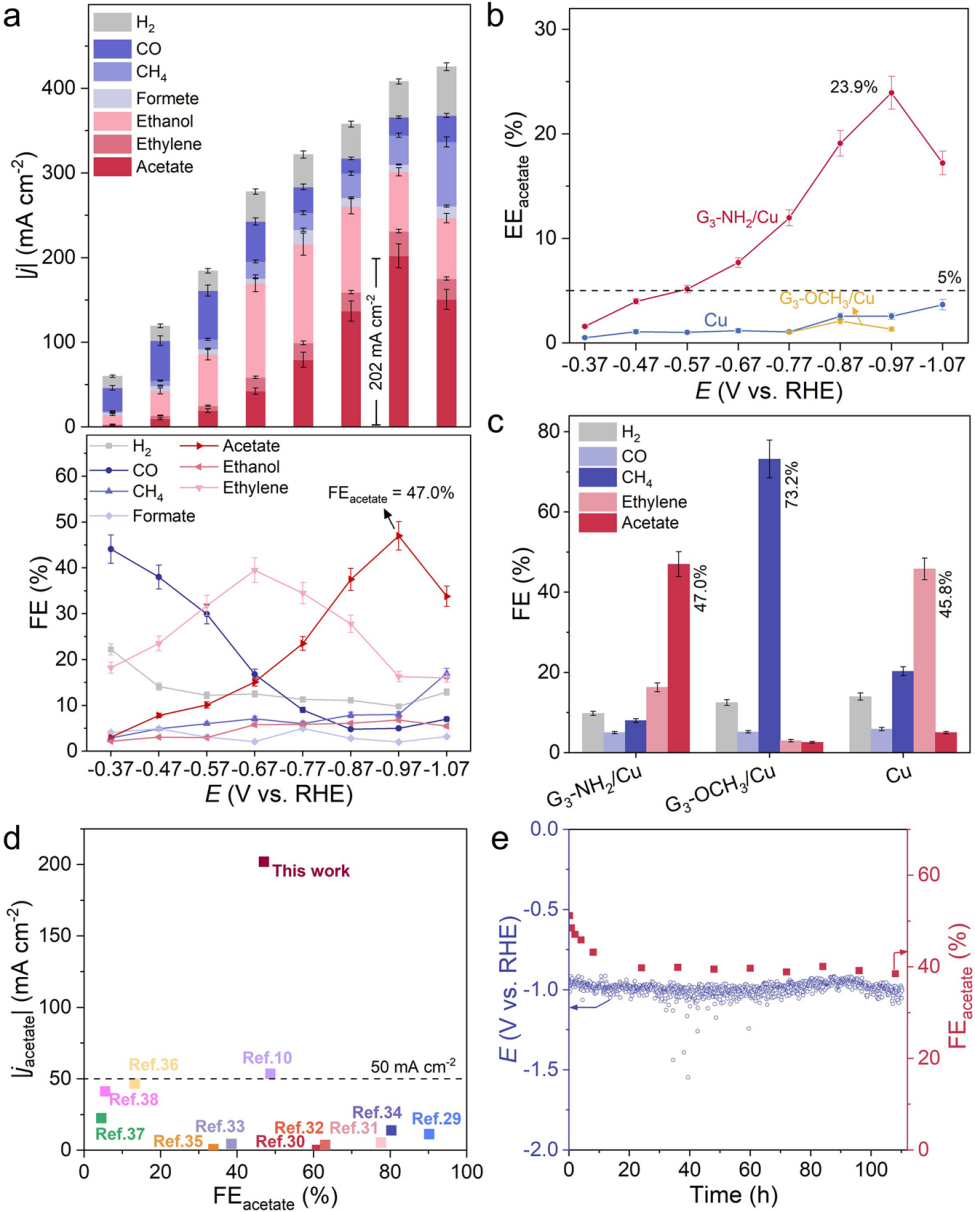
(a) Partial current densities (the upper panel) and corresponding FE values (the lower panel) of CO_2_RR products using the G_3_-NH_2_/Cu catalyst at various applied constant potentials (without ohmic correction). (b) EE values for acetate at various applied constant potentials with the G_3_-NH_2_/Cu, G_3_-OCH_3_, and Cu catalysts. (c) FE values at −0.97 V vs RHE on each catalyst. (d) Summary of |*j*_acetate_| vs FE_acetate_ of this work with other Cu-based CO_2_RR catalysts. (e) The stability obtained at a constant negative current density of −400 mA cm^–2^ (without ohmic correction). Error bars in (a–c) correspond to a mean ± standard deviation of >3 measurements.

The electrocatalytic CO_2_RR performances of G_3_-NH_2_/Cu, G_3_-OCH_3_/Cu, and pristine Cu were evaluated in flow cells (Methods in the Supporting Information). The G_3_-NH_2_/Cu catalyst not only presented the largest total current density among those three samples (Figure S8) but also showed favorable selectivity toward acetate (Figure S9 and Table S4). The peak acetate partial current density (|*j*_acetate_|) reached 202 ± 14 mA cm^–2^ at −0.97 V vs RHE, with corresponding FE_acetate_ of 47.0 ± 3.1% and a half-cell energy efficiency (EE) of 23.9 ± 1.6% (Figure 3a, b). The FEs of ethylene and CH_4_ were measured as 16.3% and 8.0%, respectively. In comparison, the G_3_-OCH_3_/Cu and Cu catalysts showed limited selectivity toward acetate (Figure 3b). For G_3_-OCH_3_/Cu, CH_4_ was the main product with a FE_CH4_ of 73.2 ± 4.7% at −0.97 V vs RHE (Figure 3c and Figure S10), while the FE values of acetate and ethylene were ∼3%. For the Cu catalyst, ethylene was the main product with FE_C2H4_ of 45.8 ± 2.7% at −0.97 V vs RHE (Figure 3c and Figure S11), and the FE values for acetate and CH_4_ were 5.0 ± 0.3% and 20.3 ± 1.1%, respectively (Figure 3c and Table S5). Our results featured one of the highest |*j*_acetate_| and excellent FE_acetate_ value, substantially exceeding the previously reported results for the CO_2_-to-acetate production (Figure 3d and Table S6).^10,29−38^ In addition, by tuning the number of amine tail chains by dendrimer generation (G_*i*_-NH_2_, *i* = 1, 2, 3, 4) for optimization of amine network (Table S7), the highest acetate selectivity in C_2_ products (i.e., FE_acetate_/(FE_acetate_ + FE_ethylene_ + FE_ethanol_)) was obtained from G_3_-NH_2_/Cu (Figure S12 and Table S8). It was observed that while the CO_2_ was captured and converted to the *CO intermediate on G_3_-NH_2_, no significant CO_2_RR performance was observed on G_3_-OCH_3_ (Figure S13). Furthermore, after >100 h of a continuous electrochemical CO_2_RR test at a constant current density of −400 mA cm^–2^, the potential of G_3_-NH_2_/Cu catalyst was stable between −0.9 to −1.1 V vs RHE, and the FE_acetate_ was retained at ∼38.5%, corresponding to ∼82% retention of its initial value (Figure 3e). The morphology (Figure S14) of G_3_-NH_2_/Cu was preserved as before electrolysis, suggesting its electrocatalytic stability.

### Mechanistic Investigation

**Figure 4:**
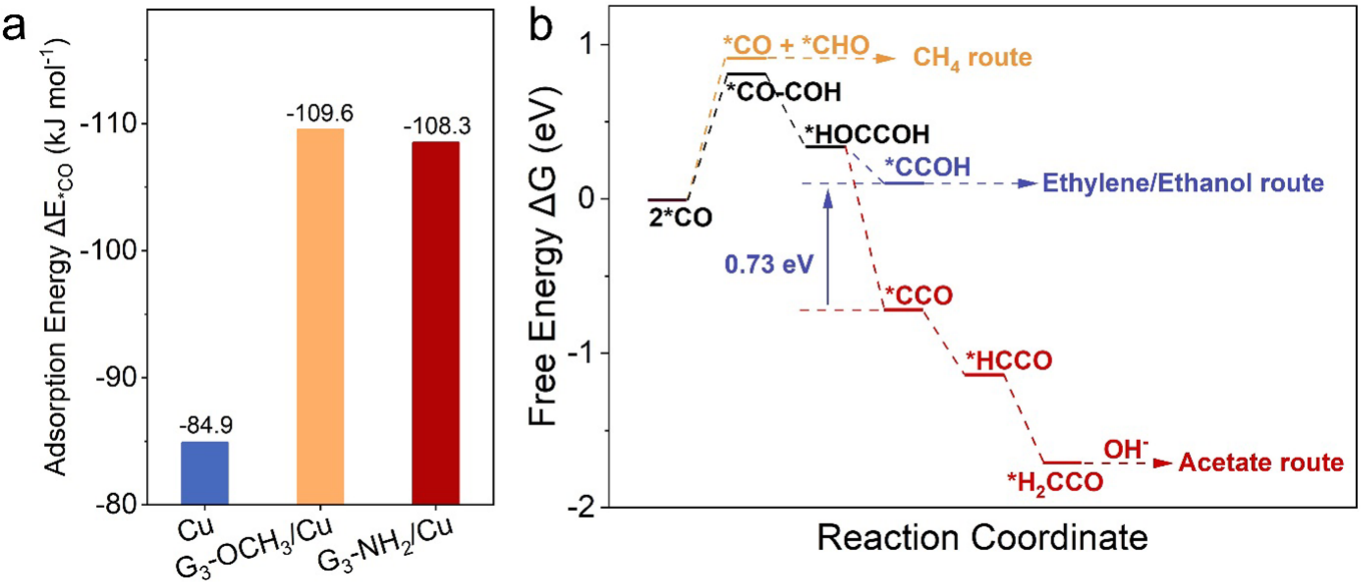
(a) Adsorption energies of *CO (ΔE_*CO_) on Cu(111), G_3_-OCH_3_/Cu(111), and G_3_-NH_2_/Cu(111) surfaces. (b) Free energy diagrams of *CO reaction pathways on the G_3_-NH_2_/Cu(111) surface.

As the electrochemical active surface areas (ECSAs) of the three electrodes evaluated by double layer capacitance (*C*_dl_)^39^ were similar (Figure S15), we hypothesized that the distinctive acetate selectivity of the G_3_-NH_2_/Cu catalyst was attributed to the high *CO coverage and high local pH. To verify this hypothesis, density functional theory (DFT) calculations were conducted to investigate the CO_2_-to-acetate pathways in those catalysts. As Cu(111) is the main exposed facet^40,41^ as well as the dominant facet in the G_3_-NH_2_/Cu, G_3_-OCH_3_/Cu, and bare Cu, we chose the Cu(111) planes for calculations (Figure S3, Figure S16, Methods in Supporting Information). The adsorption energy of *CO (ΔE_*CO_) was first compared on these models, as it affects the *CO coverage on Cu(111) surface.^7^ Compared to pure Cu(111) (−84.9 kJ mol ^–1^), the ΔE_*CO_ values on G_3_-NH_2_/Cu(111) and G_3_-OCH_3_/Cu(111) were calculated as −108.3 and −109.6 kJ mol^–1^, respectively (Figure 4a), indicating their enhanced *CO binding capabilities with surface modifications. Then, the *CO-COH coupling pathway and the competitive *CHO pathway were studied.^4,42^ The *CHO pathway was more efficient than the *CO-COH coupling in G_3_-OCH_3_/Cu(111) (Figure S17a), which led to CH_4_ formation. In contrast, both Cu(111) (Figure S17b) and G_3_-NH_2_/Cu(111) (Figure 4b) showed more efficient *CO-COH coupling than the*CHO route, indicating preferable C_2_ selectivity. The mechanism was then employed on Cu(111), where acetate is formed through the *H_2_CCO intermediate^4,43^ and competes with other C_2_ products.^40,44^

In addition, the Gibbs free energy change (Δ*G*) value of the *H_2_CCO formation step was 0.73 eV lower than *CCOH formation step on G_3_-NH_2_/Cu(111) (Figure 4b), indicating the acetate route is more favorable than the ethylene or ethanol route. As the adsorption energies of the most favorable configuration of key *CCO and *H_2_CCO intermediates on G_3_-NH_2_/Cu(111) were lower than those on Cu(111) (Figure S18 and Table S9), the *H_2_CCO species stabilized on G_3_-NH_2_/Cu(111) can promote the CO_2_-to-acetate conversion, while the main product of bare Cu(111) was C_2_H_4_, consistent with our experimental results. Thus, the functionalization of G_3_-NH_2_ on Cu(111) not only led to a strong *CO binding capability for increasing the surface *CO coverage but also enhanced the adsorption of key *H_2_CCO intermediates toward acetate production.

**Figure 5:**
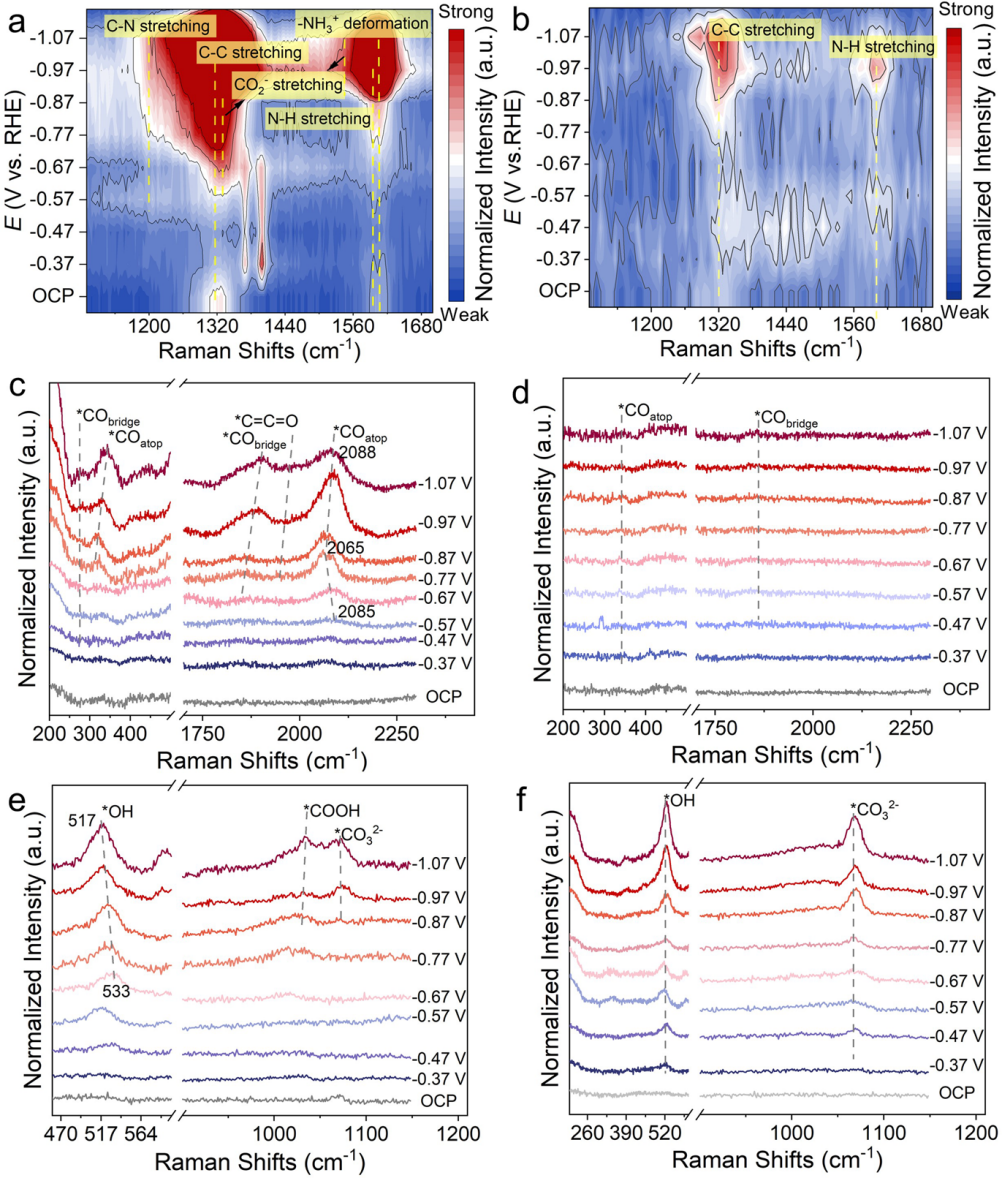
(a, b) Two-dimensional SERS spectra of (a) G_3_-NH_2_/Cu and (b) G_3_-OCH_3_/Cu. (c, d) SERS spectra of *CO vibration energy regions on (c) G_3_-NH_2_/Cu and (d) G_3_-OCH_3_/Cu. (e, f) SERS spectra of *OH and *CO_3_^2–^ vibration energy regions on (e) G_3_-NH_2_/Cu and (f) Cu.

The *in situ* electrochemical surface-enhanced Raman spectroscopy (SERS) was further performed at potentials ranging between −0.37 and −1.07 V vs RHE to confirm the contribution of *CO coverage and OH^–^ confinement on G_3_-NH_2_/Cu during CO_2_RR (Methods in Supporting Information). For G_3_-NH_2_/Cu (Figure 5a,b), the Raman peaks of C–N stretching (∼1200 cm^–1^), C–C stretching (∼1320 cm^–1^), CO_2_^–^ stretching (∼1330 and 1545 cm^–1^), N–H stretching (∼1595 cm^–1^), and N–H deformation (∼1609 cm^–1^) showed gradually increasing signals than G_3_-OCH_3_/C at more negative potentials,^11^ confirming the continued CO_2_ capture by the G_3_-NH_2_ network during CO_2_ electrolysis.^45,46^ This result was also verified with pure G_3_-NH_2_ and G_3_-OCH_3_ as electrodes for CO_2_RR at different applied potentials ranging from −0.37 to −1.07 V vs RHE (Figure S13).

In addition, the G_3_-NH_2_/Cu exhibited clearer Raman peaks located at 270–350 and 1800–2100 cm^–1^ (Figure 5c), attributed to atop-adsorbed *CO (*CO_atop_, ∼334 and 2070 cm^–1^) and bridge-adsorbed *CO (*CO_bridge_, ∼275 and 1850 cm^–1^).^47^ As shown in Figure 5c, the*CO_atop_ peaks located at 2000–2100 cm^–1^ showed a red-shift from −0.57 to −0.77 V vs RHE, indicating the *CO_atop_ vibration was affected by the vibrational Stark effect.^48^ In addition, the gradual blue shift of *CO_atop_ peaks and the appearance of *CO_bridge_ peaks at a more negative potential of −0.77 to −1.07 V vs RHE, suggesting the increased higher *CO coverage.^4,49^ Furthermore, a weak peak at ∼1970 cm^–1^ was also detected on G_3_-NH_2_/Cu, which was identified as the C=C=O stretching of an *CCO intermediate toward acetate.^47^ In comparison, for G_3_-OCH_3_/Cu (Figure 5d) and Cu (Figure S19), the *CO_bridge_ peaks were not observed and the *CO_atop_ peaks had much weaker intensities, suggesting their low surface *CO coverage.

Moreover, the Raman peaks observed on both G_3_-NH_2_/Cu and Cu showed O–H bending peaks (∼525 cm^–1^) with the increase of applied negative voltages, assigned to the *in situ* generated *OH species.^50^ Those peaks were assigned to the O–H bending modes of the surface *OH species, which are hydrogen-bonded with surrounding water molecules, as suggested by previous Raman studies.^51^ These peaks were red-shifted from 533 to 517 cm^–1^ with applied more negative potentials from −0.67 to −1.07 V vs RHE on G_3_-NH_2_/Cu (Figure 5e), implying that they were affected by the vibrational Stark effect with a Stark tuning rate of 41 ± 1.5 cm^–1^/V (Figure S20).^47,51,52^

While on the Cu surface, another C–O stretching (∼1067 cm^–1^) was observed for applied negative potentials as small as −0.47 V vs RHE (Figure 5f), corresponding to the CO_3_^2–^ species.^16,52,53^ However, this peak only became observable on G_3_-NH_2_/Cu for potentials more negative than −0.97 V vs RHE along with another peak at ∼1035 cm^–1^ corresponding to *COOH (Figure 5e).^50^ The carbonate accumulation on the Cu surface lowers the local pH^5^ and induces a higher chemical potential (Table S10), which hinders the H_2_CCO-to-acetate pathway.^54^ Taken together, the G_3_-NH_2_/Cu catalyst was allowed to confine the *in situ* generated OH^–^ on the Cu surface due to the repulsion between OH^–^ and G_3_-NH_2_/G_3_-NHCOO^–^ during the CO_2_ electroreduction. Subsequently, the confined OH^–^ reacted with H_2_CCO to form acetate, instead of reacting with CO_2_ to form CO_3_^2–^. As a result, the G_3_-NH_2_/Cu enabled a high surface *CO coverage and high local pH with *in situ* generated OH^–^ to be achieved, which facilitates the production of a *H_2_CCO intermediate toward acetate, thus boosting the CO_2_-to-acetate electrosynthesis with a peak FE_acetate_ of 47.0%, a 9.4 times improvement than that of Cu.

## Conclusion

In summary, we have demonstrated a -NH_2_-tailed, dendrimer-functionalized Cu surface aiming to enhance the CO_2_ capture and increase the local *CO concentration. The -NH_2_-rich network allowed an increase of the *CO intermediate coverage on the Cu catalytic sites, while at the same time retained the *in situ* generated OH^–^ and a high local pH environment, favoring the formation of a *H_2_CCO intermediate toward acetate. The catalyst exhibited a high CO_2_-to-acetate performance, with an FE_acetate_ of 47.0% and corresponding partial current density of 202 mA cm^–2^. Our work suggests an attractive strategy of surface molecular engineering to tune the selectivity of acetate from CO_2_RR.

## Supporting Information Available

Additional experimental and calculation details; figures of dendrimer structures, FTIR, XRD patterns, TEM and HRTEM images, EDS elemental mapping images, optical images, linear sweep voltammetry curves, representative data on gas products and liquid products distributions, partial current densities and corresponding FE values, FE graphs, CO_2_RR products distribution and total current densities, CV curves, slab models, free energy diagram, Raman spectra, vibrational frequency data; tables elemental ratios, element contents, FE values, electrochemical performances of CO_2_ reduction to acetate, absorption energies, impaction of pH condition on the reaction tendency of *H_2_CCO to acetate formation (PDF)
