# Supplementary material for: Promoting CO2 Electroreduction to Acetate by an Amine-Terminal, Dendrimer-Functionalized Cu Catalyst
Source: ACS Cent Sci. 2023 Sep 26;9(10):1905–12. doi: 10.1021/acscentsci.3c00826 (PMC10604016; doi:10.1021/acscentsci.3c00826)
Supplement: Supplementary file 1 — oc3c00826_si_001.pdf [file oc3c00826_si_001.pdf]

Supporting Information

Promoting CO_2 Electroreduction to Acetate by Amine-terminal, Dendrimer-functionalized Cu Catalyst

Li Yang,[1] Ximeng Lv,[1] Chen Peng,[1] Shuyi Kong,[2] Fuqiang Huang,[2] Yi Tang,[1] Lijuan Zhang,[1,] and Gengfeng Zheng[1,]**

[1]Laboratory of Advanced Materials, Department of Chemistry and Shanghai Key Laboratory of Molecular Catalysis and Innovative Materials, Fudan University, Shanghai 200438, China

[2]State Key Laboratory of High Performance Ceramics and Superfine Microstructure, Shanghai Institute of Ceramics, Chinese Academy of Sciences, Shanghai 200050, China.

*Corresponding authors: Lijuan Zhang, Gengfeng Zheng

Email address: zhanglijuan@fudan.edu.cn (L.Z.), gfzheng@fudan.edu.cn (G.Z.)

METHODS

Chemicals and materials. Copper sulfate pentahydrate ($CuSO_4 \cdot 5H_2O$) (≥ 99.0%), sodium sulfate anhydrous (Na_2SO_4) (≥99.0%), ethanol (≥99.7%), and acetone (≥99.5%) were all purchased from Sinopharm Chemical Reagent Co., Ltd. Potassium hydroxide (KOH) (≥95%), ethylenediamine (EDA) (≥99.0%), methyl acrylate (MA) (≥99.0%), and methanol (≥99.0%) were purchased from Aladdin Reagent (Shanghai) Co., Ltd. The impedance of deionized (DI) water was 18.2 $M\Omega \cdot cm^{-1}$.

Synthesis of G_3-NH_2 dendrimers and G_3-OCH_3 dendrimers. Poly(amidoamine) dendrimers ethylenediamine core was obtained through a typical divergent method.[1[1]] Note that all the chemical reaction processes were conducted in dark under N_2 protection and ice water bath. For the first half generation of dendrimer without repetitive unit (i.e., G_0-OCH_3), 0.3 mol of ethylenediamine initiator core was added into a 500-mL three-neck flask containing 2.0 mol of methanol under magnetic stirring for 15 minutes. Then, 2.0 mol of methyl acrylate was dropwise added to the homogeneous solution, followed by continuous stirring for 24 h. Afterwards, the solution was transferred into a 250-mL flask and rotary evaporated at 40 ℃ until the pressure in the bottle was below 0.15 kPa. The obtained G_0-OCH_3 dendrimer after purification was weighed, and the product formation yield was calculated as 99.2%.

For the first full generation of dendrimer without repetitive unit (i.e., G_0-NH_2), 0.5 mol of G_0-OCH_3 was added into a 500-mL three-neck flask containing 4.0 mol of methanol under magnetic stirring for 30 minutes, followed by dropwise addition of 3.2 mol of EDA under constant

stirring. The yellowish solution was obtained after 24 hours' reaction. Then, the above solution was transferred into a 250-mL flask and rotary evaporated at 55 ℃ until the pressure in bottle was below 0.15 kPa. The obtained G_0-NH_2 dendrimer after purification was then weighed, and the product formation yield was calculated as 99.1%.

The above procedures were repeated for half- and full-generations dendrimers, respectively. The degree of crossing-linking dendritic branches and functional terminals were controlled by the number of generation cycles, as G_i-NH_2 (i = 1, 2, 3, 4) and ester groups (G_3-OCH_3), Figure S1. The resulting different dendrimers generations in each step were purified by three-times of rotary evaporation using methanol as solvent. The resulting purified dendrimers were weighed, and the yield range was calculated as 98.5 ~ 99.4%.

Preparation of the Cu-based catalysts. All Cu-based samples comprised a carbon-paper-based gas diffusion layer (GDL) substrate or fluorine-doped tin oxide (FTO) substrate. The plating baths for G_3-NH_2/Cu, G_3-OCH_3/Cu and pure Cu were made from 10 mmol of $CuSO_4 \cdot 5H_2O$, 0.1 M Na_2SO_4 with or without 5 mmol of the as-prepared dendrimer. Galvanostatic electrodeposition on the substrate was conducted at a constant current density of −20 mA cm^{-2} until a final deposition charge of 2 C cm^{-2} was reached to generate samples with identical Cu loading. A Pt foil was used as the counter-electrode, and a saturated Ag/AgCl electrode was used as the reference electrode. The catalysts were washed with deionized DI, and ultrasonically peeled off from the substrates for further characterization.

Characterizations. X-ray diffraction (XRD) data were obtained with Bruker SMART APEX (II)-CCD (Germany). X-ray photoelectron spectroscopy (XPS) was recorded on a Perkin-Elmer PHI 5000C ESCA system (Perkin Elmer, USA). TEM, HRTEM, EDS analysis and HAADF-mapping were performed on a Titan Cubed Themis G2 300 (FEI) microscope. ^{1}H-NMR measurements were performed on a Bruker NMR600. Fourier Transform Infrared Spectroscopy (FTIR) measurement was performed on a Thermo Scientific Nicolet iN10. XANES and EXAFS data were carried out on 20BM beamline at Advanced Photon Source (APS, Argonne national laboratory, USA). Athena and Artemis software included in a standard IFEFFIT package were used to process XAS data.

CO_2 electroreduction in flow cells. Electroreduction experiments and product analysis were conducted in flow cells with an anion exchange membrane as the separator.[2] The CO_2 electroreduction activity for all catalysts were recorded by a Metrohm Autolab PGSTAT204 workstation at room temperature. Before the measurements, the cathode was purged with pure Ar for 30 min, followed by the flow of CO_2 at 30 standard cubic centimeters per minute (sccm). The 1 M KOH electrolyte flow was kept at 10 mL·min^{-1}. A gas-diffusion-layer with an IrO_2 loading of 1 mg cm^{-2} was used as the counter electrode. All cathode potentials were converted to the RHE by the Nernst equation:

$$E\text{ (vs. RHE)} = E\text{ (vs. SCE)} + 0.242\text{ V} + 0.0591 \times \text{pH} = E\text{ (vs. Ag/AgCl)} + 0.197\text{ V} + 0.0591 \times \text{pH}$$

Determination of CO_2 reduction products. The gaseous products were sampled automatically and diverted for analysis by in-line gas chromatography (Shanghai Ramiin GC 2060), equipped with both a thermal conductivity detector and a flame ionization detector to quantify H_2, CO and other alkane contents, respectively. Liquid products were quantified using fresh sample in 10% D_2O with dimethyl sulfoxide (DMSO) as an internal standard by ^{1}H-NMR spectroscopy (Bruker AVANCEAV III HD 500) via a water suppression mode.

The Faradaic efficiency (*FE*) of the liquid products can be calculated as:

$$FE = \frac{n \times F \times p \times V_{gas}}{i \times R \times T} \times 100\ \% = \frac{n \times c \times V_{liquid} \times F}{Q} \times 100\ \%$$

where n is the number of transferred electrons, F is the Faraday constant, p = 101.3 kPa, V_{gas} is the volume of gas products, i is the total current detected by the electrochemical workstation, R is the gas constant, c is the molar concentration, V_{liquid} is the volume of anode electrolyte, and Q is the quantity of applied electric charges during the CO_2 reduction.

The partial current densities (j) for products were calculated as below, where A is the geometric area of the cathode:

$$j = \frac{i \times FE}{A}$$

The energy efficiency (*EE*) for acetatewas calculated based on the half-cell in the cathode, where $E_{acetate}$ was the 0.11 V vs. RHE for CO_2 reduction reaction;[3] $E_{applied}$ was the applied potential; $FE_{acetate}$ was the Faradaic efficiency of acetate:

$$EE = \frac{(1.23 + (-E_{acetate})) \times FE_{acetate}}{1.23 + (-E_{applied})}$$

***In-situ* Raman measurements.** *In-situ* Raman measurements was conducted using a Raman spectrometer (HORIBA Jobin Yvon Inc., Model: HR Evolution) coupled with an optical microscope. The system was calibrated using a commercial Si wafer. A near-infrared laser ($\lambda = 638$ nm) was used as the excitation source. The Raman spectra were collected in the range 200–2200 cm^{-1}. A homemade spectro-electrochemical cell equipped with a Pt wire counter electrode and an Ag/AgCl reference electrode was used, with the working electrode prepared by coating the catalyst onto glassy carbon with a CO_2-pumping-1 M KOH solution. Each potential was maintained for at least 15 min before collecting the spectra.

Computational details. Density Functional Theory (DFT) calculations were performed with the *Vienna ab initio* simulation package (VASP), using the planewave basis with energy cutoff of 450 eV,[4] projector augmented wave (PAW) potentials,[5] and the generalized gradient approximation (GGA) with the Perdew−Burke−Ernzerhof (PBE) exchange-correlation functional,[6] and D3 version of Grimme's dispersion was employed for empirical dispersion correction.[7] All structures were fully relaxed by a conjugate gradient method until the residual force component was less than 0.02 eV/Å, and the convergence criterion of total energy in the self-consistent field method was set to 10^{-5} eV. The structure of Cu was obtained from JCPDS database (JCPDS No. 04-0836). The Cu (111) slab model was used to simulate metal surfaces (Figure S15), consisting of three atomic layers and 2 × 2-unit cells for the lateral dimensions, where the thickness of the vacuum was set at 15 Å to make sure that there was no superficial interaction between different layers. The Brillouin zone of the supercell was sampled by 3 × 3 × 1 uniform k-point

mesh (i.e., the Monkhorst-Pack scheme)[8]. The Gibbs free energies (ΔG) of different intermediates were calculated to characterize the CO_2RR performance on metal facets with and without organic fragment. The adsorption energies (ΔE) of the molecules were calculated by:

$$\Delta E = E_{total} - E_{substrate} - E_{mol},$$

where E_{total} and $E_{substrate}$ are the DFT energies for the system with and without adsorbate, respectively, and E_{mol} refers to the energy of a free molecule in vacuum.

The Gibbs free energy of formation (at T K) for each elementary step was calculated as:

$$\Delta G\ (T) = \Delta E_{DFT} + \Delta G_{cor}\ (T),$$

where $\Delta G_{cor}\ (T) = \Delta E_{ZPE} - T\Delta S + \Delta U(T)$ was obtained by processing frequency calculation results with VASPKIT code (version 1.2.5),[9] and ΔG_{298K} was used to construct the free energy profiles. The computational hydrogen electrode (CHE) model was also employed,[10] in which the chemical potential of $(H^+ + e^-)$ is equal to that of half a H_2 molecule with a pH correction of $k_B T\ln([H^+])$.

In addition, the effect of pH is evaluated by chemical potential based on the following equations:

$$H_2CCO\ (aq) + HO^-(aq) = Ac^-(aq),$$

$$\Delta G = G(Ac\text{-})\text{-}G(H_2CCO)\text{-}G(HO^-)\text{=}G_1\text{-}G(HO^-)\approx\mu_1 - \mu(HO^-),$$

$$\mu(HO^-) \approx \mu^\theta + RT\ln(c/c^0)\text{=}\ \mu^\theta + 2477.9\ J/mol\times \ln(c/c^0).$$

where μ refers to the corresponding chemical potential of species, and c refers to the concentration of species.

SUPPLEMENTARY FIGURES

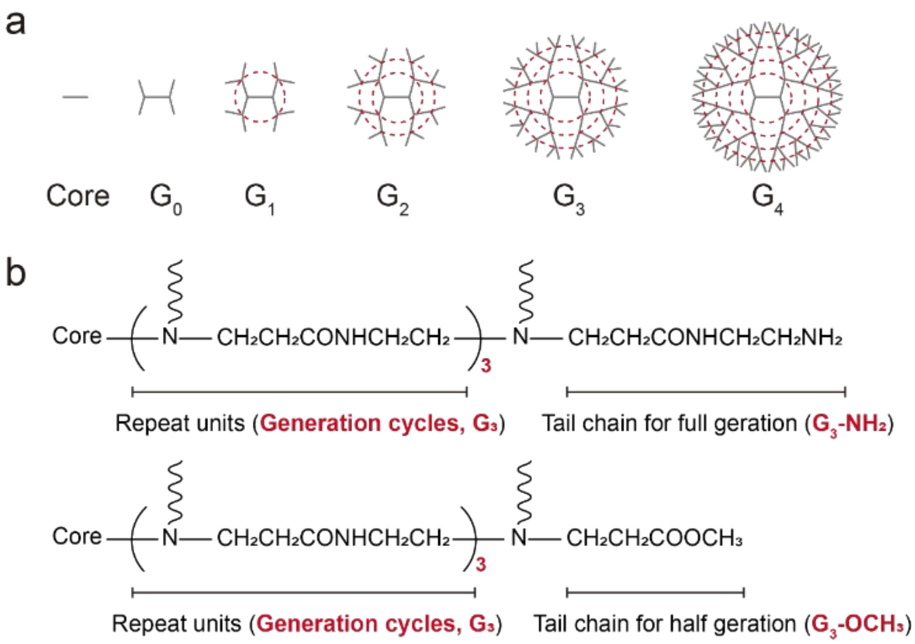

Figure S1. a) Simplified structure for poly(amidoamine) dendrimers with different functional terminus controlled by the number of generation cycles (red circles: G_i, i = 1, 2, 3, 4). b) Full generations of –NH_2-terminal dendrimers (i.e., G_3-NH_2) and half generations of -OCH_3-terminal dendrimer (i.e., G_3-OCH_3).

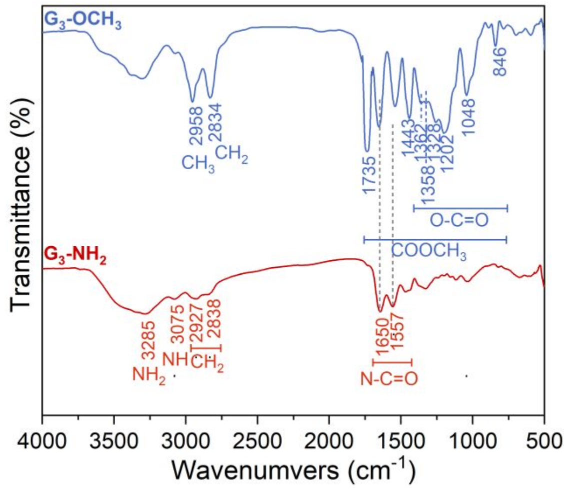

Figure S2. FITR spectra and the corresponding simplified unit structure of G_3-OCH_3 and G_3-NH_2.

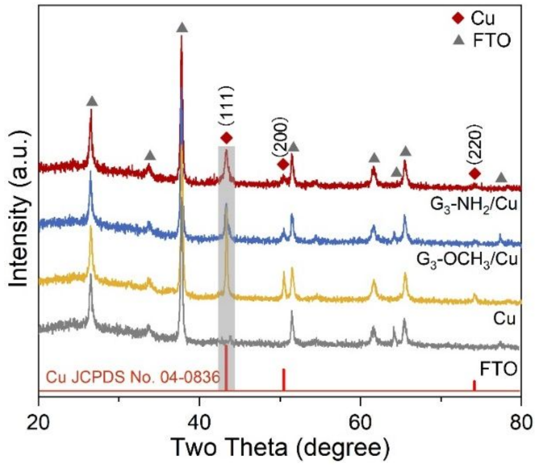

Figure S3. XRD patterns of the as-prepared catalysts assembled on FTO.

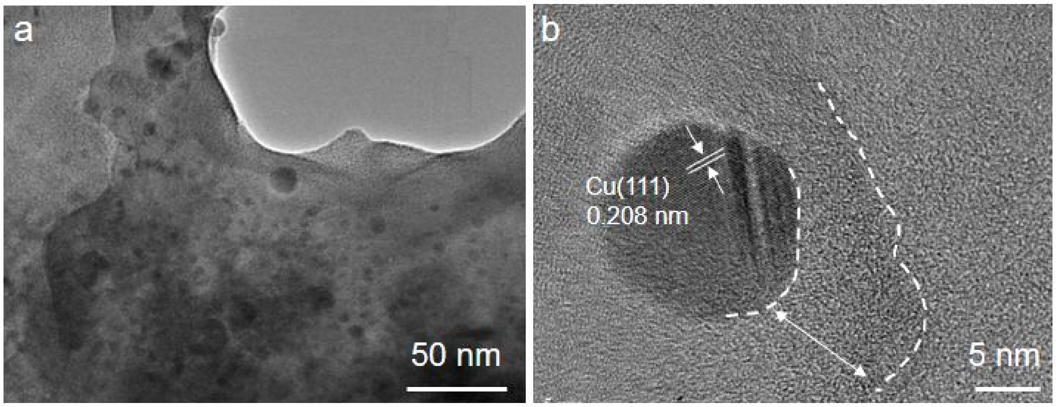

Figure S4. The a) TEM and b) the HRTEM images of G_3-OCH_3/Cu.

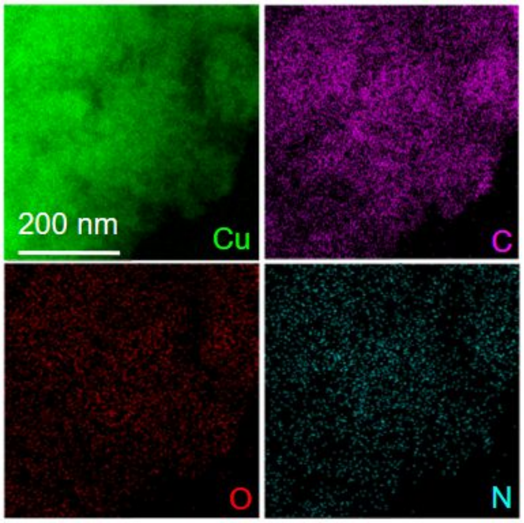

Figure S5. EDS elemental mapping images of G_3-NH_2/Cu.

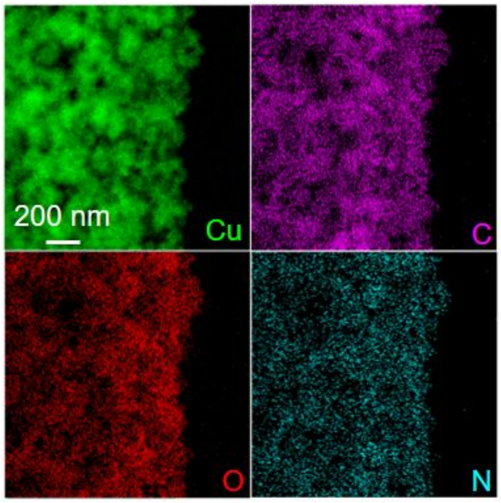

Figure S6. EDS elemental mapping images of G_3-OCH_3/Cu.

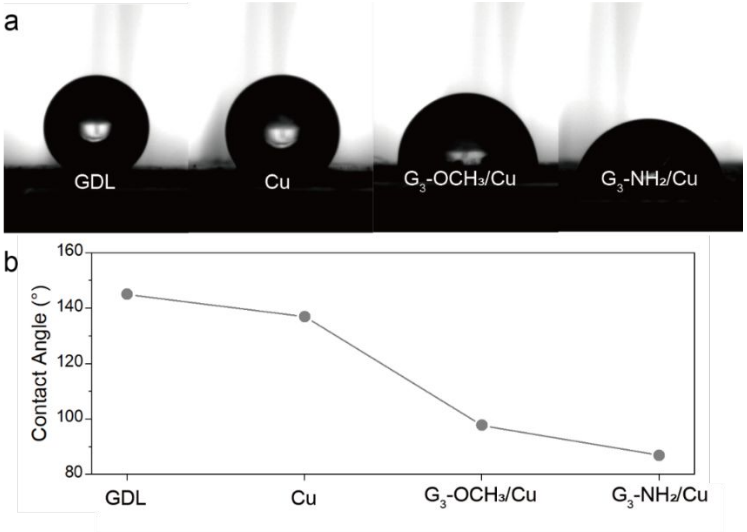

Figure S7. a) Optical images showing the wettability of the samples on a GDL, and b) the corresponding contact angle obtained using a Ramé-Hart contact angle goniometer.

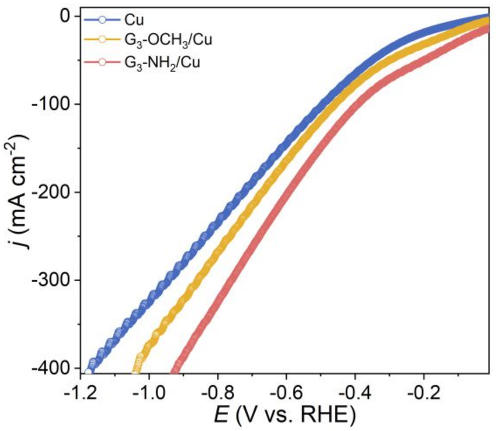

Figure S8. Linear sweep voltammetry curves of Cu, G_3-OCH_3/Cu and G_3-NH_2/Cu catalysts in 1 M KOH aqueous electrolyte in flow cells with scan rate at 50 mV s^{-1}.

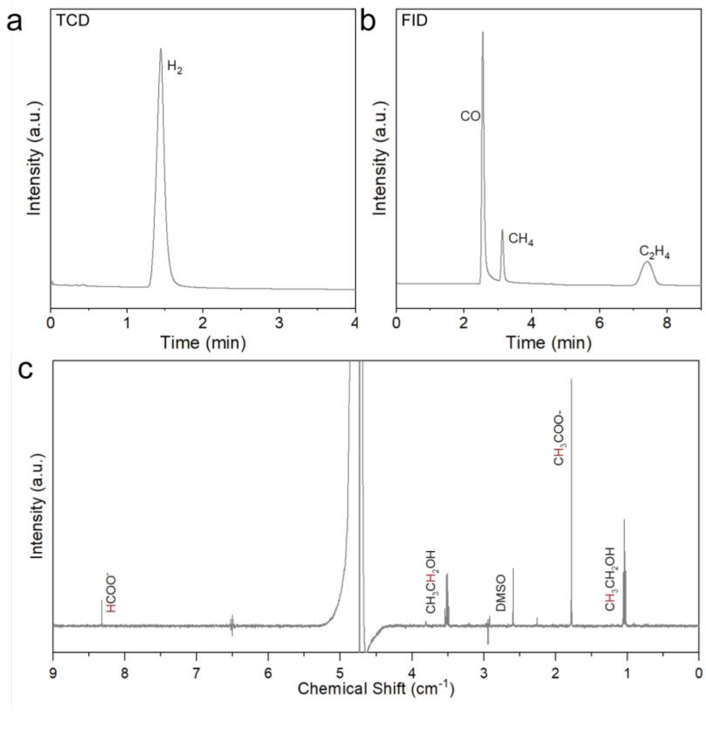

Figure S9. Representative data on gas products and liquid products distributions on G_3-NH_2/Cu. a) TCD channel and b) FID channel data for gas products at –0.97 V vs. RHE, and c) liquid products analysis at the same conditions.

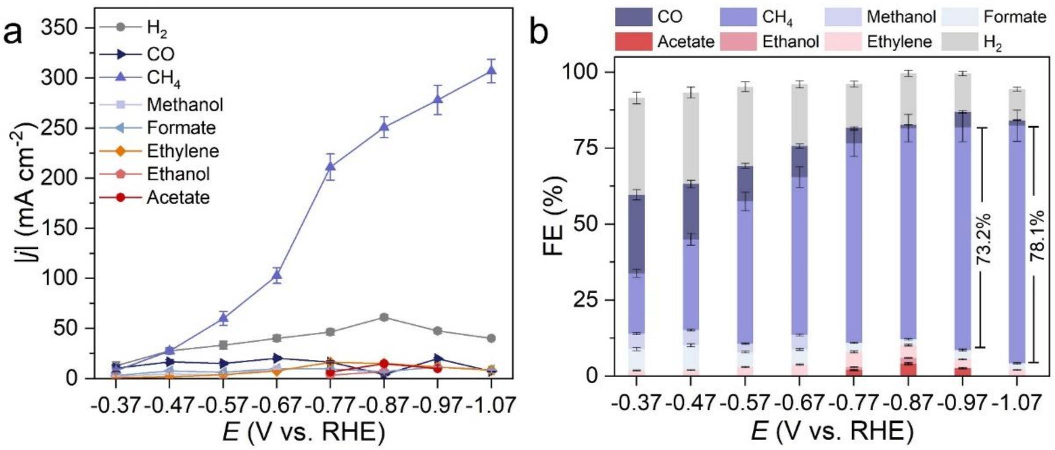

Figure S10. a) Partial current densities and b) corresponding FE values of obtained CO_2RR products using G_3-OCH_3/Cu catalyst at various applied constant potentials (without ohmic correction). Error bars correspond to mean ± standard deviation at three measurements.

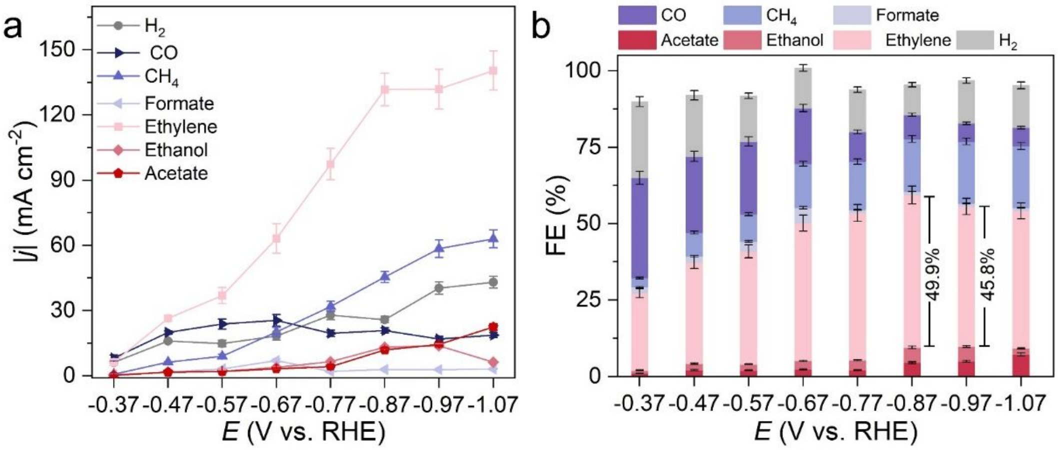

Figure S11. a) Partial current densities and b) corresponding FE values of obtained CO_2RR products using Cu catalyst at various applied constant potentials (without ohmic correction). Error bars correspond to mean ± standard deviation at three measurements.

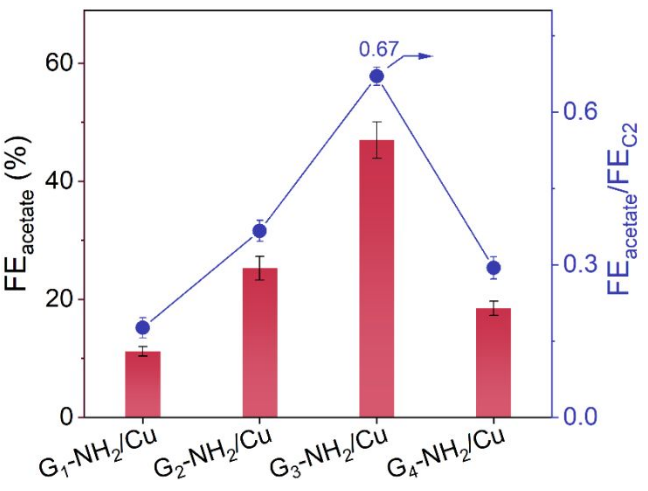

Figure S12. $FE_{acetate}$ and the ratio $FE_{acetate}/FE_{C2}$ of the four catalysts at −0.97 V vs. RHE.

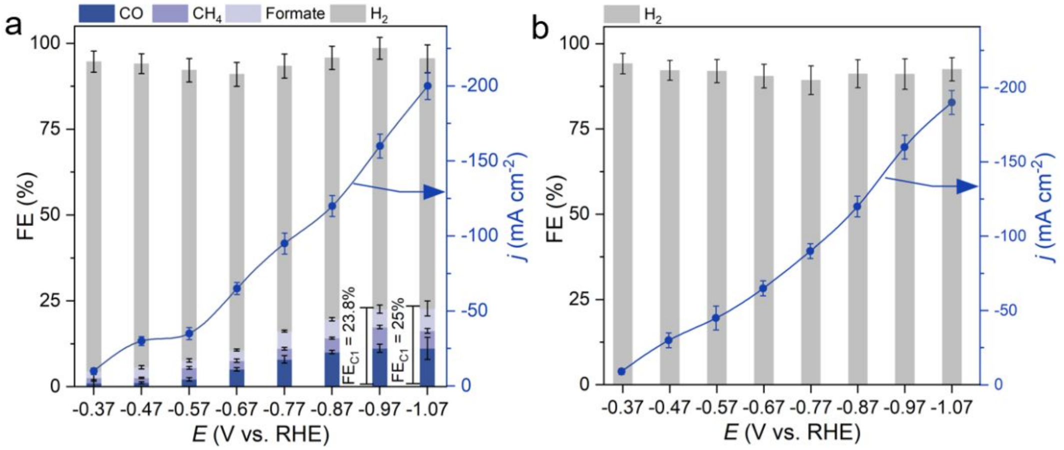

Figure S13. CO_2RR products distribution and total current densities at different applied potential ranging from –0.37 to –1.07 V vs. RHE with a) pure G_3-NH_2 and b) G_3-OCH_3 catalysts. Error bars correspond to mean ± standard deviation at three measurements. The CO_2 was captured on G_3-NH_2 and further reduced to C_1 product on G_3-NH_2 at more negative potentials (i.e., G_3-NH_2 + CO_2 + OH^- — G_3-$NHCOO^-$ + H_2O; G_3-$NHCOO^-$ + $2e^-$ + $2H_2O$ — G_3-NH_2 + CO + $3OH^-$), while no significant CO_2RR performance was observed on G_3-OCH_3.

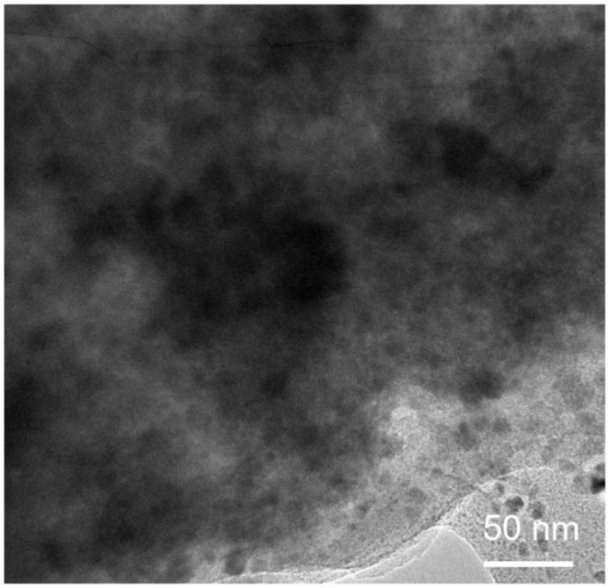

Figure S14. TEM image of G_3-NH_2/Cu after CO_2 electroreduction.

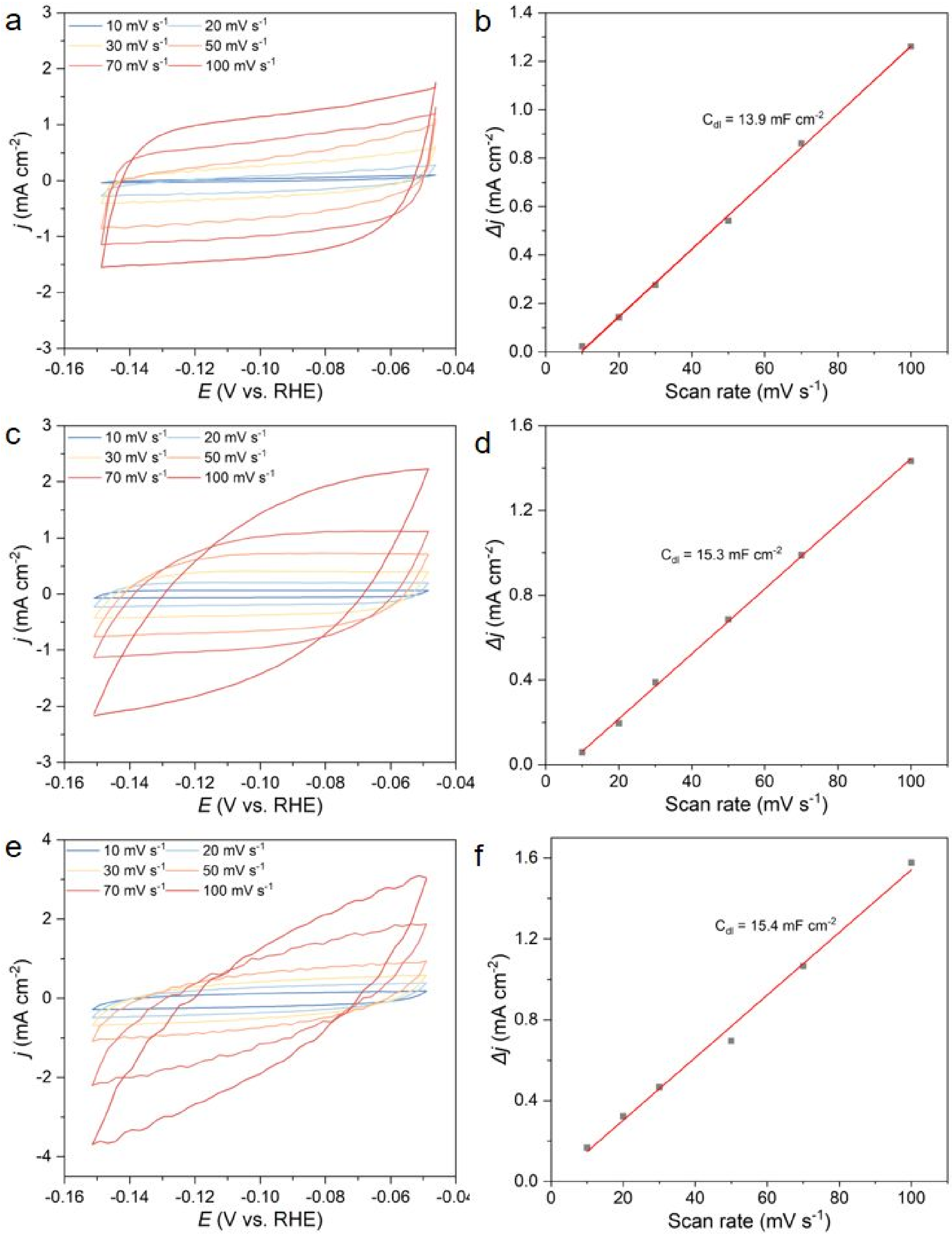

Figure S15. CV curves with different scan rates and corresponding fitting C_{dl} of a, b) Cu, c, d) G_3-NH_2/Cu and (e, f) G_3-OCH_3/Cu in 1 M KOH.

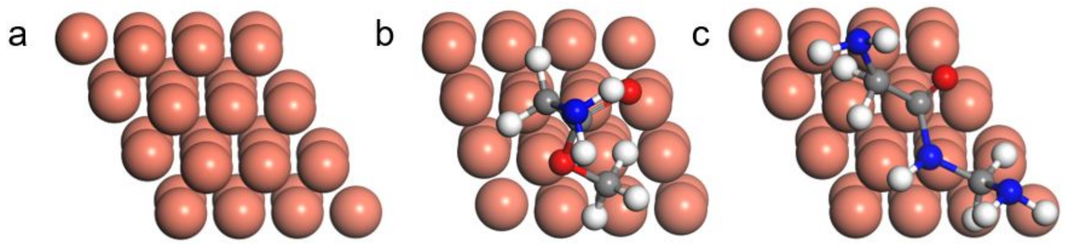

Figure S16. The slab models of a) Cu(111), b) G_3-OCH_3/Cu(111) and c) G_3-NH_2/Cu(111) for DFT calculations.

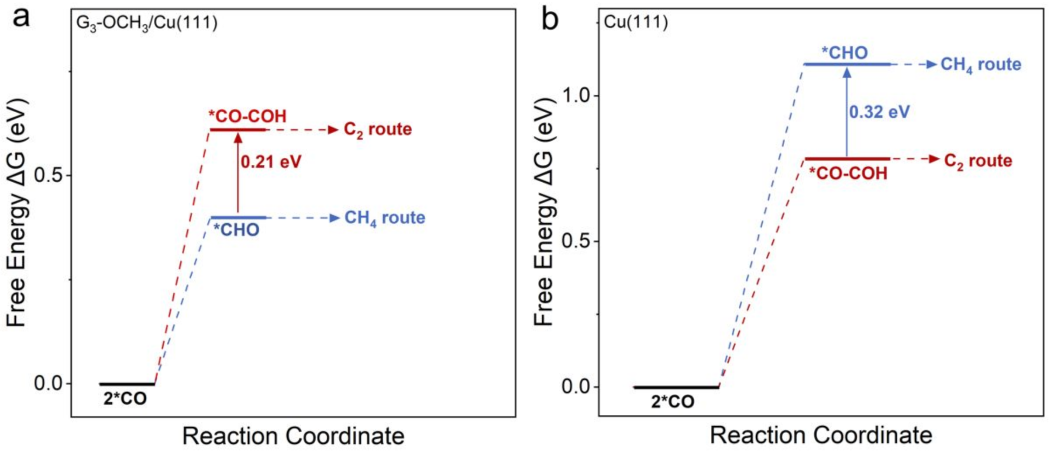

Figure S17. Free energy diagram of *CO reaction on a) G_3-OCH_3/Cu(111) and b) Cu(111) surface.

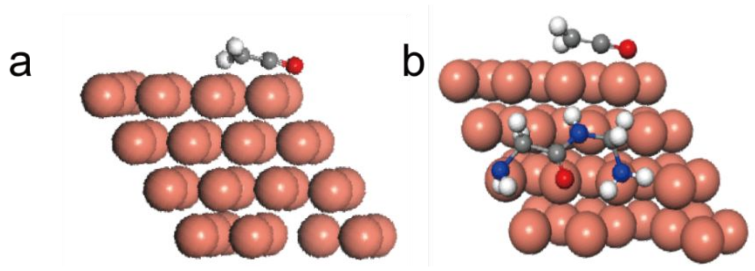

Figure S18. *H_2CCO absorbed on a) Cu(111) and b) G_3-NH_2/Cu(111).

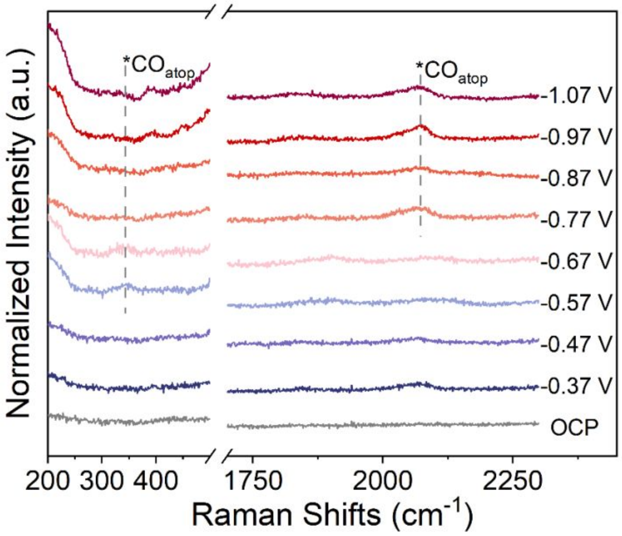

Figure S19. Potential-dependent in-situ Raman spectra obtained from Cu during CO_2 electroreduction.

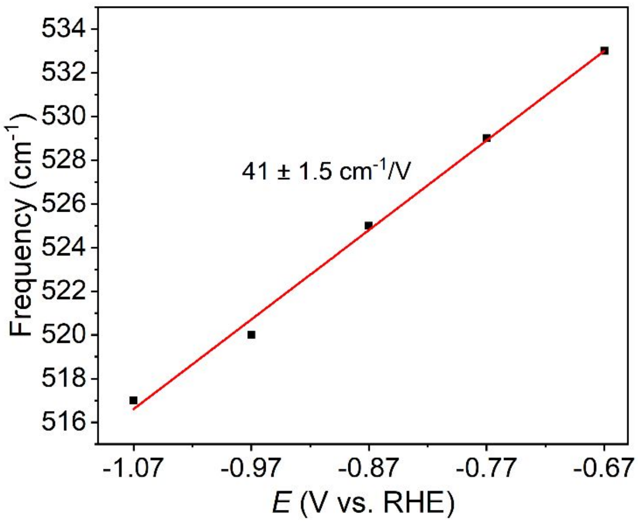

Figure S20. Vibrational frequency of *OH bond on G_3-NH_2/Cu plotted as a function of electrode potential.

Table S1. The element ratio of N/Cu in G_3-NH_2/Cu and G_3-OCH_3/Cu samples measured by EDX.

Sample	N/Cu
G_3-NH_2/Cu	0.70
G_3-OCH_3/Cu	0.32

Table S2. The element ratio of N/Cu in G_3-NH_2/Cu and G_3-OCH_3/Cu samples measured by XPS.

Sample	N/Cu
G_3-NH_2/Cu	0.72
G_3-OCH_3/Cu	0.29

Table S3. The element contents in G_3-NH_2/Cu and G_3-OCH_3/Cu samples measured by ICP.

Sample	Cu (%)
G_3-NH_2/Cu	59.53
G_3-OCH_3/Cu	62.18

Table S4. FE values for CO_2 reduction products at different applied potentials using G_3-NH_2/Cu electrocatalyst.

Potential (vs. RHE)	H_2	CO	CH_4	C_2H_4	formate	C_2H_5OH	acetate
–0.37	22.2±1.2	44.1±3.1	2.9±0.2	18.2±1.2	4.1±0.3	2.2±0.2	3.1±0.2
–0.47	14.1±0.8	38.0±2.6	4.9±0.3	23.5±1.6	4.9±0.4	3.1±0.2	7.8±0.5
–0.57	12.2±0.7	29.9±2.1	6.0±1.4	31.8±2.2	3.1±0.2	3.0±0.3	10.1±0.7
–0.67	10.5±0.7	15.8±1.1	7.1±0.5	39.5±2.7	2.1±0.2	5.8±0.4	15.1±0.9
–0.77	11.3±0.6	9.0±0.6	6.0±0.5	34.5±2.3	5.0±0.4	5.9±0.5	23.5±1.5
–0.87	11.1±7	4.8±0.3	7.9±0.6	27.8±1.9	2.8±0.2	6.1±0.3	37.5±2.4
–0.97	9.8±0.5	5.0±0.3	8.0±0.6	16.3±1.1	2.0±0.2	6.8±0.4	47.0±3.1
–1.07	12.9±0.7	7.0±0.5	17.0±1.1	16.0±1.0	3.2±0.3	5.5±0.4	33.8±2.2

Table S5. FE values for CO_2 reduction products using different electrocatalysts at –0.97 V vs. RHE.

Electrocatalyst	H_2	CO	CH_4	C_2H_4	formate	C_2H_5OH	acetate
G_3-NH_2/Cu	9.8±0.5	5.0±0.3	8.0±0.5	16.3±1.1	2.0±0.2	6.8±0.4	47.0±3.1
G_3-OCH_3/Cu	12.5±0.7	5.2±0.3	73.2±4.7	3.0±0.3	3.0±0.5	0	2.6±0.2
Cu	14.0±0.9	5.9±0.4	20.3±1.1	45.8±2.7	1.0±0.2	4.8±0.3	5.0±0.3

Table S6. Summary of electrochemical performances of CO2 reduction to acetate.

Catalysts	Electrolytes	Potential (vs. RHE)	FE (%) /acetate	|*j*| (mA cm^{-2}) /acetate	Ref.
G_3-NH_2/Cu	1 M KOH	–0.97 V	47.0%	202	This work
Fe/N-C	0.05 M $KHCO_3$	–0.5 V vs. Ag/AgCl)	60.9%	0.22	11
NDD/Si RA	0.5 M $NaHCO_3$	–1.0 V	77.6%	~5.4	12
Mn-corrole-CP	0.1 M phosphate buffer (pH 6)	–0.67 V	63%	3.75	13
Cu-Cu_2O	0.1 M KCl	–0.4 V	38.5%	4.5	14
Mo_8@Cu/TNA	1 M KOH	–1.13 V	48.68%	53.5	15
PcCu-TFPN	0.1 M KOH	–0.8 V	90.2%	11.3	16
Cu1/BN-C_{30}	0.01 M LiI in 25 mol% [Emim]BF_4 and 75 mol% water	–2.2 V (vs. Ag/Ag^+)	80.3%	13.9	17
HMMP Cu_3Zn	0.1 M $KHCO_3$	–1.0 V (vs. RHE)	33.8%	~0.74	18
CuZn/CuZnA l_2O_4	2 M KOH	–1.15 V	13.2%	46.7	19
Cu CF	1 M KOH	–1.05 V	4.5%	22.5	20
6.2% Pd–Cu	0.5 M K_2SO_4-H_2SO_4 aqueous solution (pH 2.0)	at a current density of 750 mA cm^{-2}	5.5%	41.25	21

Table S7. The element ratio of N/Cu measured by XPS for Cu samples assembled with dendrimers of different generation cycles (G_i-NH_2, i = 1, 2, 3, 4).

Sample	N/Cu
G_1-NH_2/Cu	0.20
G_2-NH_2/Cu	0.58
G_3-NH_2/Cu	0.72
G_4-NH_2/Cu	1.71

Table S8. FE values for H_2 and CO_2 reduction C_2 products using different electrocatalysts at –0.97 V vs. RHE.

Electrocatalyst	H_2	C_2H_4	C_2H_5OH	acetate	Total C_2 products
G_1-NH_2/Cu	13.3±0.8	42.5±2.9	9.8±0.3	11.2±0.4	54.3
G_2-NH_2/Cu	10.4±0.6	28.2±1.9	15.5±0.6	25.3±1.7	54.4
G_3-NH_2/Cu	9.8±0.6	16.3±1.1	6.8±0.4	47.0±3.1	63.7
G_4-NH_2/Cu	17.3±1.1	26.2±1.8	18.2±1.1	18.5±1.4	45.8

Table S9. The adsorption energies (E_{ad}) of the most favorable configuration of *CCO species and *H_2CCO species on Cu(111) in the presence and absence of G_3-NH_2. All the adsorption energies are reference to the one on Cu(111).

Surface	E_{ad}'(*CCO) (eV)	E_{ad}'(*H_2CCO) (eV)
Cu(111)	–4.04	0.04
G_3-NH_2/Cu(111)	–4.22	–0.35

Table S10. The impaction of pH condition on the reaction tendency of *H_2CCO to acetate formation (i.e., from the perspective of chemical potential difference Δμ).

Condition	$[OH^-]$ ($mol·L^{-1}$)	γ	RTlna_{OH-} ($kJ·mol^{-1}$)	Δμ (versus pH=14, eV)
pH=12	0.01	0.926	−11.6	−0.12
pH=13	0.1	0.81	−6.3	−0.07
pH=14	1	-	0	0

REFERENCES

(1) Tomalia, D. A.; Baker, H.; Dewald, J.; Hall, M.; Kallos, G.; Martin, S.; Roeck, J.; Ryder, J.; Smith, P. A new class of polymers: starburst-dendritic macromolecules. *Polym J* **1985**, *17*, 117–132.

(2) Verma, S.; Lu, X.; Ma, S. C.; Masel, R. I.; Kenis, P. J. A. The effect of electrolyte composition on the electroreduction of CO_2 to CO on Ag based gas diffusion electrodes. *Phys. Chem. Chem. Phys.* **2016**, *18*, 7075–7084.

(3) Nitopi, S.; Bertheussen, E.; Scott, S. B.; Liu, X.; Engstfeld, A. K.; Horch, S.; Seger, B.; Stephens, I. E. L.; Chan, K.; Hahn, C.; Nørskov, J. K.; Jaramillo, T. F.; Chorkendorff, I. Progress and perspectives of electrochemical CO_2 reduction on copper in aqueous electrolyte. *Chem. Rev.* **2019**, *119*, 7610–7672.

(4) Kresse, G. Efficient iterative schemes for ab initio total-energy calculations using a plane-wave basis set. *Phys. Rev. B* **1996**, *54*, 11169–11186.

(5) Kresse, G. From ultrasoft pseudopotentials to the projector augmented-wave method. *Phys. Rev. B* **1999**, *59*, 1758–1775.

(6) Perdew, J. P.; Burke, K.; Ernzerhof, M. Generalized gradient approximation made simple. *Phys. Rev. Lett.* **1996**, *77*, 3865–3868.

(7) Grimme, S.; Ehrlich, S.; Goerigk, L. Effect of the Damping function in dispersion corrected density functional theory. *J Comput Chem* **2011**, *32*, 1456–1465.

(8) Monkhorst, H. J.; Pack, J. D. Special points for brillouin-zone integrations. *Phys. Rev. B* **1976**, *13*, 5188–5192.

(9) Wang, V.; Xu, N.; Liu, J.-C.; Tang, G.; Geng, W.-T. VASPKIT: A user-friendly interface facilitating high-throughput computing and analysis using VASP code. *Comput Phys. Commun.* **2021**, *267*. 108033.

(10) Nørskov, J. K.; Rossmeisl, J.; Logadottir, A.; Lindqvist, L. Origin of the overpotential for oxygen reduction at a fuel-cell cathode. *J. Phys. Chem. B* **2004**, *108*, 17886–17892.

(11) Genovese, C.; Schuster, M. E.; Gibson, E. K.; Gianolio, D.; Posligua, V.; Grau-Crespo, R.; Cibin, G.; Wells, P. P.; Garai, D.; Solokha, V.; Krick, C. S.; Valasco-Velez, J. J.; Ampelli, C.; Perathoner, S.; Held, G.; Centi, G.; Arrigo, R. Operando spectroscopy study of the carbon dioxide electro-reduction by iron species on nitrogen-doped carbon. *Nat. Commun.* **2018**, *9*, 935.

(12) Liu, Y.; Chen, S.; Quan, X.; Yu, H. Efficient electrochemical reduction of carbon dioxide to acetate on nitrogen-doped nanodiamond. *J. Am. Chem. Soc.* **2015**, *137*, 11631–11636.

(13) De, R.; Gonglach, S.; Paul, S.; Haas, M.; Sreejith, S. S.; Gerschel, P.; Apfel, U. P.; Vuong, T. H.; Rabeah, J.; Roy, S.; Schöfberger, W. Electrocatalytic reduction of CO_2 to acetic acid by

a molecular manganese corrole complex. *Angew. Chem. Int. Ed.* **2020**, *59*, 10527–10534.

(14) Zhu, Q.; Sun, X.; Yang, D.; Ma, J.; Kang, X.; Zheng, L.; Zhang, J.; Wu, Z.; Han, B. Carbon dioxide electroreduction to C_2 products over copper-cuprous oxide derived from electrosynthesized copper complex. *Nat. Commun.* **2019**, *10*, 3851.

(15) Zang, D.; Li, Q.; Dai, G.; Zeng, M.; Huang, Y.; Wei, Y. Interface engineering of Mo_8/Cu heterostructures toward highly selective electrochemical reduction of carbon dioxide into acetate. *Appl. Catal. B* **2021**, *281*, 119426.

(16) Qiu, X. F.; Huang, J. R.; Yu, C.; Zhao, Z. H.; Zhu, H. L.; Ke, Z.; Liao, P. Q.; Chen, X. M. A Stable and conductive covalent organic framework with isolated active sites for highly selective electroreduction of carbon dioxide to acetate. *Angew Chem. Int. Ed.* **2022**, *61*, e202206470.

(17) Sun, X.; Zhu, Q.; Kang, X.; Liu, H.; Qian, Q.; Ma, J.; Zhang, Z.; Yang, G.; Han, B. Design of a Cu(i)/C-doped boron nitride electrocatalyst for efficient conversion of CO_2 into acetic acid. *Green Chem.* **2017**, *19*, 2086–2091.

(18) Su, X.; Sun, Y.; Jin, L.; Zhang, L.; Yang, Y.; Kerns, P.; Liu, B.; Li, S.; He, J. Hierarchically porous Cu/Zn bimetallic catalysts for highly selective CO_2 electroreduction to liquid C_2 products. *Appl. Catal. B* **2020**, *269*, 118800.

(19) Zhang, Z.-Y.; Tian, H.; Bian, L.; Liu, S.-Z.; Liu, Y.; Wang, Z.-L. Cu-Zn-based alloy/oxide interfaces for enhanced electroreduction of CO_2 to C_{2+} products. *J. Energy Chem.* **2023**, *83*, 90–97.

(20) Fang, M.; Wang, M.; Wang, Z.; Zhang, Z.; Zhou, H.; Dai, L.; Zhu, Y.; Jiang, L. Hydrophobic, ultrastable $Cu^{\delta+}$ for robust CO_2 electroreduction to C_2 products at ampere-current levels. *J. Am. Chem. Soc.* **2023**, *145*, 11323–11332.

(21) Xie, Y.; Ou, P.; Wang, X.; Xu, Z.; Li, Y. C.; Wang, Z.; Huang, J. E.; Wicks, J.; McCallum, C.; Wang, N.; Wang, Y.; Chen, T.; Lo, B. T. W.; Sinton, D.; Yu, J. C.; Wang, Y.; Sargent, E. H. High carbon utilization in CO_2 reduction to multi-carbon products in acidic media. *Nat. Catal.* **2022**, *5*, 564–570.
